# An improved method for physician-certified verbal autopsy reduces the rate of discrepancy: experiences in the Nouna Health and Demographic Surveillance Site (NHDSS), Burkina Faso

**DOI:** 10.1186/1478-7954-9-34

**Published:** 2011-08-04

**Authors:** Maurice Yé, Eric Diboulo, Louis Niamba, Ali Sié, Boubacar Coulibaly, Cheik Bagagnan, Jonas Dembélé, Heribert Ramroth

**Affiliations:** 1 Centre de Recherche en Santé de Nouna, Burkina Faso, PO BOX 02 Nouna, Burkina Faso; 2 University of Heidelberg, Institute of Public Health, Heidelberg, Germany

**Keywords:** Verbal autopsy, cause of death, discrepancy, concordance, Nouna, Burkina Faso

## Abstract

**Background**

Through application of the verbal autopsy (VA) approach, trained fieldworkers collect information about the probable cause of death (COD) by using a standardized questionnaire to interview family members who were present at the time of death. The physician-certified VA (PCVA), an independent review of this questionnaire data by up to three physicians trained in VA coding, is currently recommended by the World Health Organization (WHO) and is widely used in the INDEPTH Network. Even given its appropriateness in these contexts, a large percentage of causes of death assigned by VAs remains undetermined. As physicians often do not agree upon a final COD classification, there remains substantial room to improve the standard VA method, potentially leading to a reduction in physician discordance in COD coding.

**Methods**

We present an extension of the current method of PCVA and compare it to the standard WHO-recommended procedure. We used VA data collected in the Nouna Health and Demographic Surveillance Site (NHDSS) between 2009 and 2010 using a locally-adapted version of an INDEPTH standard verbal autopsy questionnaire. Until 2009, physicians in the NHDSS followed the WHO method (Method 1). As an extension of Method 1, starting in 2010, the use of a panel of physicians was added to the coding process in the case where a third physician's final conclusions resulted in an undetermined COD (Method 2). Two independent samples of VA questionnaires were compared for the year 2009 (using Method 1) and the year 2010 (using Method 2).

**Results**

The WHO-recommended method used for 2009 yielded a high level of undetermined CODs, where the final coding was "undetermined" in 50.8% of all questionnaires due to disagreement among participating physicians (Method 1). By introducing a panel of physicians in 2010 for cases where the principal physicians disagreed on the cause of death, the revised method significantly reduced the proportion of undetermined CODs to 1.5% (Method 2).

**Conclusions**

As the extended method of PCVA significantly improved the accuracy of the VA procedure, we suggest the adoption of this method for those countries where alternatives like computer-based VA coding are not available. Based on the results of our study, further research should be pursued.

## Background

Verbal autopsy (VA) is a technique used to determine the cause of death by asking caregivers, friends, or family members about signs and symptoms exhibited by the deceased in the period before death. It is usually done by trained fieldworkers using a standardized questionnaire that collects details on signs, symptoms, complaints, and any medical history or events prior to death [1].

The World Health Organization (WHO) recommends the use of verbal autopsy to measure specific causes of death [2,3]. The purpose of verbal autopsy is to describe the causes of death at the community or population level where limited or no vital registration is completed with medical certificates. Indeed, medically certified cause of death data are available for less than one-third of the more than 57 million deaths occurring worldwide annually. The majority of deaths lacking such data are from developing countries [4]. Information about cause of death is essential for public health planning, priority setting, monitoring, and evaluation, but the collection of such information in countries with incomplete or no vital registration systems remains a substantial challenge [5]. Reliable data on cause-specific mortality is also needed by countries to keep track of progress toward the Millennium Development Goals [3,6].

The use of physician-certified verbal autopsies (PCVA) is common in the majority of developing countries, as well as for Health and Demographic Surveillance Sites (HDSS) that are members of International Network for the Demographic Evaluation of Populations and Their Health in Developing Countries (INDEPTH) [6]. Within the INDEPTH Network, 36 HDSS in 20 countries regularly use VA to assess cause of death [6]. However, the data collection tools are not yet harmonized, which has led to substantial variability in the coding process across sites [7,8]. Recently, there have been several attempts to introduce alternative methods such as a computer-based verbal autopsy coding method (InterVA) to replace the PCVA approach [6]. This probability-based method was tested in several settings [9-11]. However, the results still show some discrepancies in comparison to PCVA results [12]. Few studies are available on the use of different physician coding methods that produce better results. In contrast, the study by Joshi and colleagues comparing results involving multiple coders versus one single coder suggest that advantages attained from the multiple-coding system remain limited [13]. However, in this study, the approaches for cause of death assignment used either a panel of expert physicians or involved two or more physician coders who independently reviewed the data to arrive at a final diagnosis [14,15]. The method proposed in this paper was tested with the intention to build on former approaches, such as those presented in the study by Joshi et al [13].

In this study we compare the usual WHO-recommended PCVA procedure with a locally-adapted method that incorporates the use of a panel of physicians after a discrepancy among three physician coders arises.

## Methods

### Study area

**Figure 1:**
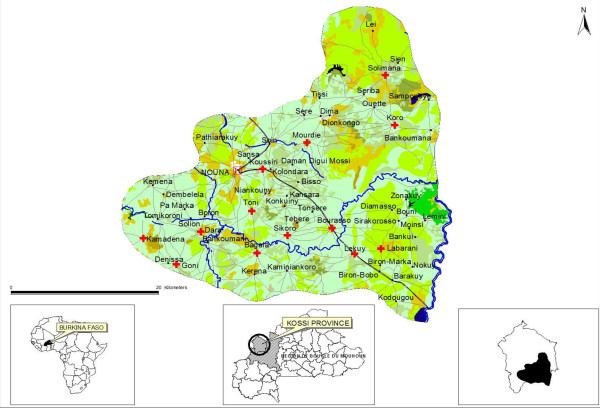
**Map of NHDSS**.

The Nouna Health and Demographic Surveillance Site (NHDSS) has existed since 1992 and is in the rural western part of Burkina Faso (Figure 1). It currently covers 58 villages and one semi-urban town and covers a population of about 85,000 inhabitants. The NHDSS is part of Kossi province, which consists primarily of a rural population of multi-ethnic groups. The predominant activity is subsistence farming and cattle keeping. The region is a dry orchard savannah and has a sub-Sahelian climate, which is characterized by a hot climate with short rainy season lasting from June to September with rainfall varying between 400 to 1000 millimeters. The vegetation is mainly scattered short trees. The mean temperature varies from 26°C to 34°C, often reaching 40°C in April, the hottest period [11].

The NHDSS is a member of the INDEPTH Network, a global network of HDSSs with the aim of conducting longitudinal health and demographic evaluation of populations in low- and middle-income countries [16]. The health facilities within the NHDSS consist of one secondary care facility (the district hospital) and 14 primary health centers. The NHDSS has been used as a sampling frame for numerous studies in the fields of clinical research, epidemiology, health economics, and health-systems research. Nouna has a functional vital event registration system, which allows collecting data continuously on pregnancies, births, deaths, and migration [17].

### The VA questionnaire

The Nouna questionnaire covers background characteristics of the deceased using structured filter questions on specific signs and symptoms experienced by the deceased up to the point of death. Additionally, a narrative section provides an opportunity to describe conditions not covered in the structured questions (see Additional file 1). Although the questionnaire is written in French, interviews with the HDSS population are performed by trained fieldworkers who translate the content into local languages. The Dioula language is the most spoken local language, but several other languages are common, such as Bwamu, Moore, and Fulfulde.

Verbal autopsy questionnaire data are collected every four to five months at the household level by interviewers. They are then coded by physicians familiar with the 10^th ^revision of the WHO International Classification of Diseases codes (ICD-10).

We used the ICD-10 adopted in 1994 by the World Health Assembly. Its main use here is to classify causes of mortality as recorded at the registration of death. The ICD-10 also covers a conceptual framework of definitions, standards, and methods that have been closely linked and developed along with the classifications themselves. A restricted list based on ICD-10 has been used for the final physician coding (see Additional file 2).

### Physicians' coding organization

The VA coding sessions were organized locally by gathering 12 physicians working in the district hospital with an average working experience as general practitioners of four years. One of these physicians with detailed public health background guided the coding process. All physicians had good knowledge of patient management covering the areas of general medicine, care for pediatric inpatients, care for HIV patients, and basic gynecological and obstetrical care for women. Nevertheless, the panel sought opinion from external specialists in the area of interest when required. Based on the number of available physicians, the panel consisted of three to four members. An agreement upon a given cause of death was only reached when two out of three members (66%) or three out of four members (75%) of the panel arrived at a consensus. Thus the panel coding process was more than majority-based and required that more than 50% of the panel members come up with the same cause of death. The cause was then ascribed to the final cause of death. The panel overwhelmingly agreed to classify the cause of death as undetermined if the available VA information did not allow them to make a final decision.

### Study design

This study was designed as a comparative study using two methods of PCVA to ascertain causes of death respectively on two independent samples of VA questionnaires collected in 2009 and 2010.

The first sample, from 2009, was coded using the WHO-recommended method (Method 1). The second sample, from 2010, was coded using the extended method (Method 2).

#### Coding methods

Method 1: As recommended by WHO [3], two experienced local physicians interpret the answers to the questionnaire and independently determine the most probable cause of death. In the case of disagreement, a third physician is consulted. The cause of death is attributed only if supported by at least two physicians using ICD-10.

Method 2: In 2010, Method 1 was extended using a panel of physicians in the case of a coding discordance between referee physicians.

**Figure 2:**
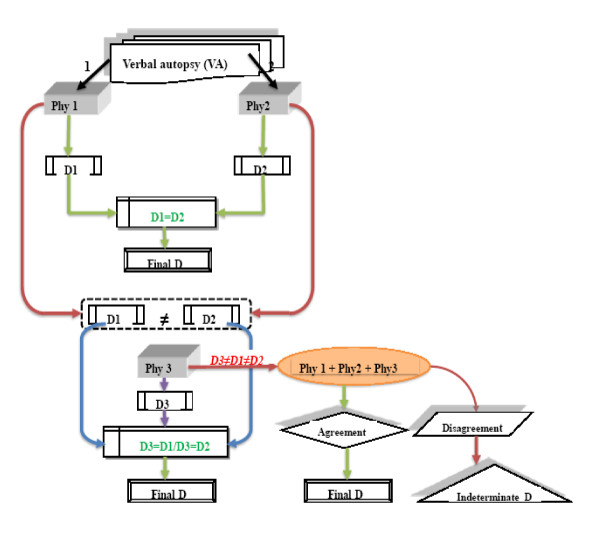
**Verbal autopsy coding procedure**. Phy: physician, D: diagnosis.

The VA coding procedure has been combined in a stepwise process shown in Figure 2.

### Verbal autopsy data collection

**Figure 3:**
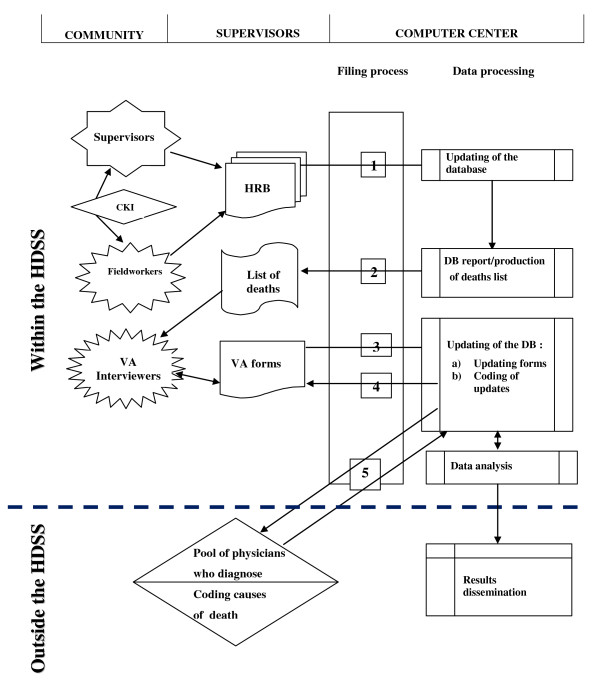
**Verbal autopsy flowchart**. The filing process steps are as follows: 1) Events processing for data entry; 2) Processing output of VA interviews; 3) Processing of VA forms for data entry; 4) Process followed in the case of problems reported on the VA forms; 5) Processing of VA coding for data entry; HRB: compound registration book, DB: database, CKI: community key informants.

Two key actors are generally involved in the process of VA data collection. Since the creation of Nouna HDSS, the event of death is registered in an active reporting system using community reporters, called community key informants (CKIs). Overall, 58 CKIs (one per village) report deaths occurring within households. Afterward, an assigned village interviewer collects information on the death. The trained field staff who visit households with a registered death have no medical background. As described above, they conduct the interview with the caregivers or relatives, translating the French VA questionnaire into the local language. The interview usually takes place several months after the event with the person who assisted the deceased before the death. Figure 3 presents the VA data collection flow chart in Nouna describing the interaction between fieldworkers and the community.

### Quality control

Quality control is ensured by several checking mechanisms put in place at different stages of the data collection process. Whenever inconsistencies in collected information do not allow for a final diagnosis, a second interview is done by a field supervisor for consistency. Independently, the interview process at the household level is closely followed up by village supervisors in a random manner. At the data-entry level, attention is given to the attributed codes to reduce errors of coding.

### Statistical method used

The concordance rate was obtained for each method by taking the total number of VAs coded where there is agreement among physician coders over the total number of VAs coded.

The proportion test for two independent samples was applied to compare the proportions of undetermined cause of death achieved using the different methods.

## Results

### Verbal autopsy data coded

Out of 1,256 deaths collected over the study period, 640 were coded in 2009 using the first coding method (WHO), while 616 deaths were coded in 2010 using the locally-adapted method.

### Agreement between physician coders

Out of 640 deaths coded in 2009 using the WHO method, there was an agreement on 315 diagnoses. This represents a concordance rate of 49.2% for the first method. Applying the same procedure to the VA records of 2010, agreement could be achieved for only 219 records, resulting in a preliminary concordance rate of 35.6%.

Involvement of the physician panel increased agreement on the final cause of death to 607 diagnoses. Thus, the latter method yielded a concordance rate of 98.5% among physician coders, given the two stages of analysis. With additional involvement of a physician panel in the case of disagreement among the principal coders, the discrepancy among physician coders could thus substantially be reduced to less than 1.5%.

The results of the proportion test showed that the proportions of undetermined causes of death achieved by the two methods were significantly different (p value < 0.0001). The study shows a significant reduction in the percentage of undefined causes of deaths.

### Predominant cause-specific mortality fraction

**Figure 4:**
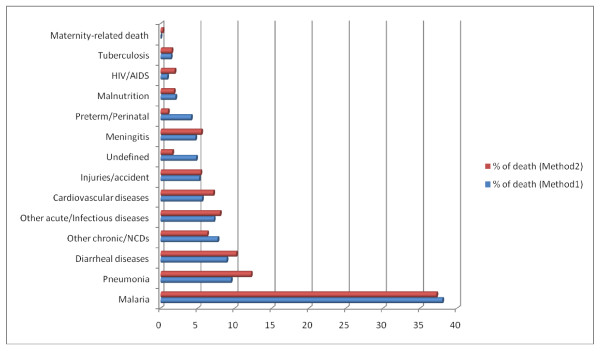
**Comparative cause-specific mortality fractions of Methods 1 and 2**.

Our findings indicate that malaria is the leading cause of death, 37.3% in 2009 and 37.9% in 2010, of total deaths registered (Figure 4). Here the undetermined CODs are not displayed, assuming that the non coded CODs follow the same pattern as the coded CODs. Malaria is followed by pneumonia and diarrheal diseases. Figure 4 shows similar patterns in deaths using the two methods.

## Discussion

Our findings provide evidence that the choice of verbal autopsy coding method has a highly significant impact on the results of PCVA. It indicates that the improvement of an empirical method of PCVA, like the WHO-recommended method [3], through use of a physician panel in case of COD-coding disagreements, leads to a high reduction of the proportion of undetermined causes of death. Although this method of panel coding necessitates additional resources and time for physician coders as compared to the standard coding procedure described by Soleman et al [5], and especially to the single-coding procedure described by Joshi et al [13], it brings a large improvement in the existing methods determining the probable causes of death.

The findings of Fottrell et al [9] support our results, as an initial agreement of 60% among two physicians was shown to increase to more than 80% when a review is done by one additional physician. Thus, our findings are in stark contrast to those of Joshi and colleagues who suggest reducing coding to one physician only [9]. We cannot exclude the fact that the quality of coding might depend on the VA questionnaire used per HDSS site, as discussed in several INDEPTH meetings over the past five years. The current VA questionnaire available through WHO/INDEPTH tries to overcome limitations of existing VA questionnaires, offering separate versions for different age groups and providing comparability over different countries. Thus, NHDSS has moved to the updated WHO/INDEPTH questionnaire in 2011. For coding, the Nouna site uses the restricted classification list suggested during the INDEPTH Meeting in Uganda in 2008, comparable with other HDSS sites.

On one hand, our data possibly suggest that the new multicoding system of deaths doesn't necessarily affect the mortality pattern, although it results in changes in the proportion of deaths within the different groups of leading causes of death. Undeniably, the suggested method is more time-consuming and costly, but it is also more efficient. However, this is the first time that such a panel discussed the questionable cases. In summary, the procedure might be especially helpful in HDSS sites where high rates of undetermined CODs are observed.

The use of automated Bayesian models to assign the most likely causes of death tested by Byass [18] are currently under investigation in the Nouna HDSS. The main gains achieved from this method are a reduction in time and cost needed to complete the coding process. Additionally, as the model doesn't involve different physicians over time or in different countries, it aims to provide comparable results within HDSS sites over time and across different HDSS sites. However, its use is still limited to certain sites and the result comparison with PCVA approaches still shows some discrepancies in comparison to the PCVA results [12].

However, given that the computer-based probability approach to VA interpretation is designed to overcome the weakness of physicians' reviews, preliminary results are promising, but are not fully convincing [9-11]. At present, the main problem in choosing an optimal method of coding for VA is that no gold standard is available and comparison among various methods remains limited. Currently, both methods might profit from comparing their results with those by the other method.

For resource-poor settings, a reliable and affordable method of VA coding remains a necessity, as mortality data remain important to guide decision-makers for health planning purposes. While waiting to scale up use of the computer-based model, the improved WHO method proposed in our study could be applied as an alternative method for coding, as it offers a good rate of concordance among physician coders.

Despite verbal autopsy being a useful tool in determining causes of death, the method has some limitations. Previous studies note these shortcomings in detail [2,5,9,13]. Because verbal autopsy is based on data collected through an interview process, and based on signs and symptoms exhibited, it is subject to recall bias and misreporting. Physicians have different experiences and knowledge in coding that could lead to different interpretations of the diagnosis [5,9].

While PCVA has some well-known limitations [4], the shortcomings of the tool are known and quantifiable. These deficiencies, however, should not prevent countries requiring information on causes of death from benefiting from the use of VA when no practical alternative for obtaining these data exists. Few studies are available on the use of different physician coding methods that produce better results. However, the approaches for cause of death assignment most commonly used either a panel of expert physicians or two or more physician coders who independently review the data and arrive at a diagnosis [14,15]. Despite its acknowledged limitations [13], PCVA is still considered the best possible method to get cause of death estimates in areas where vital events registrations systems are limited or not available.

Cohen's kappa as a measure of agreement couldn't be applied, as both samples used here were independent (years 2009 and 2010) and the coding was done independently in a blind manner by different physicians. Given this constraint, we focused only on the comparison of concordance rate between the two samples. This approach to analysis allowed us to attain a simple but effective measure for the improvement of the extended coding method.

There is a need for further study to confirm our findings in other settings. This will have the advantage of adopting a unique method for HDSS sites within the INDEPTH Network and to some extent to other sites outside the network interested in more accurate physician-certified verbal autopsy coding methods.

## Conclusions

Verbal autopsy remains essential to capture and determine probable cause of death, especially in the context of low-income countries like Burkina Faso, where it is estimated that roughly 75% of deaths occur at home [19]. The VA process has the ability to contribute substantially by informing policymakers on real mortality data and allowing countries to monitor trends toward attainment of Millennium Development Goals, in particular those related to maternal and child health outcomes. The advantage of involving a physician panel in the coding process as suggested here is obvious, as it allows coding of an additional 50% of VAs. Importantly, this method promotes interactive discussions among physicians involved in the coding process, similar to what physicians are already doing during their clinical presentations on patients. Thus, the panel method provides a framework for scientific discussion among physicians, allowing everyone to update their knowledge.

Our study presents an alternative method of PCVA that substantially reduces the proportion of undetermined causes of death and therefore contributes to the death codification. We also aim to advocate for harmonization in the PCVA process, while encouraging the validation of the computer-based method of death coding.

## List of abbreviations

**CKI**: community key informants; **COD**: cause of death; **ICD**: International Classification of Diseases; **INDEPTH**: International Network for the Demographic Evaluation of Populations and Their Health in Developing Countries; **NHDSS**: Nouna Health and Demographic Surveillance Site; **PCVA**: physician-certified verbal autopsy; **VA**: verbal autopsy; **WHO**: World Health Organization

## Competing interests

The authors declare that they have no competing interests.

## Authors' contributions

MY: Participated in the study design, data analysis and interpretation, drafted the paper, and coordinated the manuscript revision process. ED: Participated in the study design, performed statistical analysis, and helped write the paper. LN: Participated in the data analysis and interpretation and helped write the paper. AS: Participated in data analysis and interpretation and helped write the paper. BC: Participated to data analysis and interpretation and helped write the paper. CB: supervised data entry, performed data analysis, and helped write the paper. JD: Supervised data collection and contributed to data acquisition, analysis, and interpretation. HR: Contributed to the study design, data analysis and interpretation, and helped draft and revise the manuscript. All authors read and approved the final manuscript.

## Supplementary Material

Additional file 1 **The VA questionnaire used in the NHDSS**. Click here for file

Additional file 2 **The restricted cause of death list based on ICD-10 used for the final physician coding**. Click here for file
