# Supplementary material for: An improved method for physician-certified verbal autopsy reduces the rate of discrepancy: experiences in the Nouna Health and Demographic Surveillance Site (NHDSS), Burkina Faso
Source: Popul Health Metr. 2011 Aug 4;9:34. doi: 10.1186/1478-7954-9-34 (PMC3160927; doi:10.1186/1478-7954-9-34)
Supplement: Additional file 1 — The VA questionnaire used in the NHDSS. [file 1478-7954-9-34-S1.PDF]

ANNEX 1

QUESTIONNAIRE STANDARDISE D'AUTOPSIE VERBALE

CENTRE DE RECHERCHE EN SANTE DE NOUNA

HISTOIRE DES EVENEMENTS AYANT CONDUIT AU DECES

S'AGIT-IL D'UN ACCIDENT ? **OUI** **NON**

Si oui : *Faire la chronologie des événements qui se sont déroulés après l'accident ayant conduit au décès sur la partie "Histoire des symptômes et des traitements".*

Si non : *Répondez d'abord aux questions suivantes :*

Le premier symptôme de la maladie mortelle: ....

Durée de la maladie mortelle jusqu'au décès:
(H=heures; J=jours; S=semaines; M=mois; A=années): |__|__| |__|

Est-ce qu'il y avait une autre maladie avant ce premier symptôme (Y=oui; N=non): |__|

si oui: laquelle: ....

durée de cette autre maladie
(J=jours; S=semaines; M=mois; A=années): |__|__| |__|

Maintenant, remplissez "l'histoire des symptômes et des traitements".

HISTOIRE DES SYMPTOMES ET DES TRAITEMENTS:

En cas de symptômes particuliers faire décrire ou mimer par le répondant. Dans chaque cas préciser les traitements reçus (éventuellement en utilisant le registre de la formation sanitaire) et l'ordre de succession des événements.

....

....

....

....

....

....

....

....

....

....

....

....

....

....

....continuer au verso en cas de besoin.

Maintenant, remplissez seulement les parties sur les symptômes, que le décédé avait (selon les parents).

FIEVRE OU CORPS CHAUD **OUI** **NON**

Durée (H=heures; J=jours; S=semaines; M=mois; A=années): |__|__| |__|

Quand cela a-t-il commencé (date): |__|__| |__|__| |__|__|

Quand cela s'est-il terminé (date): |__|__| |__|__| |__|__|

Fièvre très forte (F)/moyenne (M): |__|

Fièvre intermittente (I)/continue (C): |__|

Transpirations (Y=oui; N=non): |__|

Frissons (Y=oui; N=non): |__|

Nivaquine prise au cours de la fièvre (Y=oui; N=non): |__|

+ si oui: précisez, combien de comprimés au total: |__|__|

Injections reçues pour cette fièvre (Y=oui; N=non): |__|

+ si oui: où: .... nombre |__|

quand la première fois (date): |__|__| |__|__| |__|__|

Durée du traitement (H=heures; J=jours; S=semaines): |__|__| |__|

DIARRHEE OU DYSENTERIE **OUI** **NON**

Durée (H=heures; J=jours; S=semaines; M=mois; A=années): |__|__| |__|

Début (date): |__|__| |__|__| |__|__| Fin (date): |__|__| |__|__| |__|__|

Selles très liquides (Y=oui; N=non): |__|

Selles glaireuses/muqueuses/sanglantes (Y=oui; N=non): |__|

Combien de selles par jour ? |__|__|

Bouche sèche ou souvent soif (Y=oui; N=non): |__|

Yeux enfoncés (Y=oui; N=non): |__|

Mains creusées (Y=oui; N=non): |__|

Enfants de moins de 2 ans : Fontanelle déprimée (Y=oui; N=non): |__|

VOMISSEMENTS **OUI** **NON**

Durée (H=heures; J=jours; S=semaines; M=mois; A=années): |__|__| |__|

Quand au cours de la maladie ? (D=début; C=au cours; F=vers la fin; T=tout au long): |__|

Combien de fois par jour ? |__|__|

Couleur des vomissements:
(J=jaune; V=vert; R=rouge; N=noir (atre); A=alimentaire; X=autres) |__|

Vomissements en jet (Y=oui; N=non): |__|

CRISES CONVULSIVES, RAIDEUR DU CORPS **OUI** **NON**

RAIDEUR DU COU, COU REJETE EN ARRIERE **OUI** **NON**
Continuez avec les questions secondaires
si au moins une des deux dernières réponses est "OUI"

Nombre de crises par jour: |__|__|

Durée de chaque crise (M=minutes; H=heures; J=jours): |__|__| |__|

Quand au cours de la maladie (D=début; C=au cours; F=vers la fin; T=tout au long): |__|

Spasme, mouvement brusque et incontrôlé (Y=oui; N=non): |__|

Cou rejeté en arrière (Y=oui; N=non): |__|

Jambes tendues/fléchies (Y=oui; N=non): |__|

Bras tendus/fléchis (Y=oui; N=non): |__|

Poings fermés (Y=oui; N=non): |__|

Bouche fermée/crispée (ne peut plus manger) (Y=oui; N=non): |__|

Cris ou pleurs au cours de la crise (Y=oui; N=non): |__|

Emission d'urines pendant la crise (Y=oui; N=non): |__|

Morsure de la langue (Y=oui; N=non): |__|

Hypersalivation (bavait beaucoup) (Y=oui; N=non): |__|

Enfant de moins de 2 ans: Fontanelle bombée (Y=oui; N=non): |__|

S'AGIT-IL D'EPILEPSIE ? **OUI** **NON**

Date de la première crise : |__|__| |__|__| |__|__|

Etait-il soigné (préciser où): ....

DIFFICULTES A RESPIRER **OUI** **NON**

Durée (H=heures; J=jours; S=semaines; M=mois; A=années): |__|__| |__|

Quand cela a-t-il commencé ? |__|__| |__|__| |__|__|

Quand cela s'est-il terminé ? |__|__| |__|__| |__|__|

Respiration rapide (Y=oui; N=non): |__|

Respiration difficile (Y=oui; N=non): |__|

Respiration bruyante/sifflante (Y=oui; N=non): |__|

Ailes du nez palpitantes (Y=oui; N=non): |__|

Peau rentrait entre les côtes (Y=oui; N=non): |__|

TOUX **OUI** **NON**

Durée (H=heures; J=jours; S=semaines; M=mois; A=années): |__|__| |__|

Quand cela a-t-il commencé ? |__|__| |__|__| |__|__|

Quand cela s'est-il terminé ? |__|__| |__|__| |__|__|

Toux la nuit (Y=oui; N=non): |__|

Crachait après la toux (Y=oui; N=non): |__|

+ si oui: crachats purulents ou nauséabonds (Y=oui; N=non): |__|

Crachait du sang (Y=oui; N=non): |__|

Vomissait après la toux (Y=oui; N=non): |__|

Perdait sa respiration (suffoque) en toussant (Y=oui; N=non): |__|

S'AGIT-IL DE LA COQUELUCHE ? **OUI** **NON**

Combien de jours après le début de la toux est-il décédé ? |__|__|

Un autre enfant de la concession avait-il la même
coqueluche à la même période ? (Y=oui; N=non): |__|

+ si oui: préciser où il a été contaminé: ....

BOUTONS **OUI** **NON**

Durée (H=heures; J=jours; S=semaines; M=mois; A=années): |__|__| |__|

Quand cela a-t-il commencé: |__|__| |__|__| |__|__|

Quand cela s'est-il terminé: |__|__| |__|__| |__|__|

Où sur le corps ? ....

Où ont-ils commencé : ....

Ont-ils apparus ensemble (E) ou les uns après les autres (A): |__|

Boutons plats (P) / saillants (S): |__|

Boutons grands (G) / petits (P): |__|

Contenaient-ils de l'eau (Y=oui; N=non): |__|

Ont-ils cicatrisé avant le décès (Y=oui; N=non): |__|

La peau a-t-elle desquamée (Y=oui; N=non): |__|

Se grattait le corps (Y=oui; N=non): |__|

S'AGIT-IL DE LA ROUGEOLE ? **OUI** **NON**

Combien de jours après l'éruption des boutons est-il décédé ?: |__|__|

S'il n'a pas eu de boutons, préciser les symptômes qui vous ont permis de reconnaître la rougeole:

....

Un autre enfant de la concession avait-il la rougeole
à la même période (Y=oui; N=non): |__|

+ si oui: préciser où il a été contaminé: ....

PLAIES, LESIONS, BRULURES **OUI** **NON**

Localisation: ....

Plaie infectée (Y=oui; N=non): |__|

SAIGNEMENTS **OUI** **NON**

Localisation: ....

Combien de fois : |__|__|

Quantité (F=faible; M=moyenne; I=importante): |__|

Quand au cours de la maladie (D=début; C=au cours; F=vers la fin; T=tout au long): |__|

OEDEMES, CORPS ENFLE, VENTRE GONFLE **OUI** **NON**

Durée (H=heures; J=jours; S=semaines; M=mois; A=années): |__|__| |__|

Quand cela a-t-il commencé: |__|__| |__|__| |__|__|

Quand cela s'est-il terminé: |__|__| |__|__| |__|__|

Douleur en urinant (Y=oui; N=non): |__|

COULEUR ANORMALES DES URINES **OUI** **NON**

Préciser la couleur: (J=jaune; B=blanchâtre; R=rouge; C=claire) |__|

Quand au cours de la maladie: (D=début; C=au cours; F=vers la fin; T=tout au long) |__|

COULEUR ANORMALES DES SELLES **OUI** **NON**

Préciser la couleur: (J=jaune; V=vert; R=rouge; N=noir(âtre); A=alimentaire; X=autres) |__|

Quand au cours de la maladie (D=début; C=au cours; F=vers la fin; T=tout au long): |__|

MAL AUX YEUX, COULEUR ANORMALES DES YEUX **OUI** **NON**

Préciser: ....

Quand au cours de la maladie (D=début; C=au cours; F=vers la fin; T=tout au long): |__|

Yeux rouges et larmoyants (Y=oui; N=non): |__|

S'AGIT-IL D'UN DECES DE NOUVEAU-NE ? **OUI** **NON**
(dans les 4 semaines après la naissance)

S'AGIT-IL D'UN DECES PENDANT LA GROSSESSE ? **OUI** **NON**

S'AGIT-IL D'UN DECES APRES ACCOUCHEMENT (max. 42 j) ? **OUI** **NON**

+ si oui: préciser le nombre de jours après accouchement |__|__|

Si la réponse à toutes les trois dernières questions est " non", veuillez continuer à la page 10.

LA GROSSESSE

Durée de la grossesse (mois): |__|__|

La mère a-t-elle été malade durant la grossesse (Y=oui; N=non): |__|

+ si oui: préciser: ....

Mains enflées (Y=oui; N=non): |__|

Jambes enflées (Y=oui; N=non): |__|

Visage enflé (Y=oui; N=non): |__|

Difficulté à marcher (Y=oui; N=non): |__|

Saignements pendant la grossesse (Y=oui; N=non): |__|

+ si oui: quand: (D=début; C=au cours; F=vers la fin; T=tout au long): |__|

A-t-elle été soignée au cours de la grossesse (Y=oui; N=non): |__|

+ si oui: où: ....

comment: ....

A-t-elle eu un régime alimentaire particulier (Y=oui; N=non): |__|

+ si oui: lequel: ? ....

A-t-elle été à la visite prénatale (Y=oui; N=non): |__|

+ si oui: où: ....

A-t-elle reçu une injection (Y=oui; N=non): |__|

+ si oui: où: ....

La femme a-t-elle été malade au cours des grossesses précédentes (Y=oui; N=non): |__|

+ si oui: préciser: ....

ACCOUCHEMENT

Lieu de l'accouchement: ....

L'accouchement a-t-il présenté des difficultés ou des complications (Y=oui; N=non): |__|

+ si oui: préciser: ....

Naissance multiple (jumeaux/triplets) (Y=oui; N=non): |__|

Durée du travail (H=heures; J=jours): |__|__| |__|

Tête venue la première (Y=oui; N=non): |__|

Le placenta est-il venu normalement et en entier (Y=oui; N=non): |__|

Le cordon s'est-il infecté ou sentait-il mauvais (Y=oui; N=non): |__|

La femme a-t-elle été soigné longtemps (Y=oui; N=non): |__|

+ si oui: combien de temps: (M=minutes; H=heures; J=jours) |__|__| |__|

couleur du sang: (R=rouge; N=noir(âtre)) |__|

A-t-elle eu des sueurs ou les mains froides (Y=oui; N=non): |__|

ETAT DE L'ENFANT

L'enfant est-il: né vivant (V)/mort-né(M)/non-né(N)/avorté(A) |__|

L'enfant présentait-il une malformation (Y=oui; N=non): |__|

+ si oui: préciser laquelle: ....

Etait-il normal (N) trop gros (G) trop maigre (M): |__|

Etait-il normal (N) trop grand (G) trop petit (P): |__|

Avait-il une tête trop grosse (Y=oui; N=non): |__|

A-t-il crié rapidement après la naissance (Y=oui; N=non): |__|

A-t-il respiré normalement après la naissance (Y=oui; N=non): |__|

Urinait-il normalement (Y=oui; N=non): |__|

Déféquait-il normalement (Y=oui; N=non): |__|

A-t-il tété (Y=oui; N=non): |__|

A-t-il tété jusqu'au décès (Y=oui; N=non): |__|

A-t-il reçu un sérum antitétanique (Y=oui; N=non): |__|

+ si oui: où: .... quand: |__|__| |__|__| |__|__|

S’AGIT-IL D’UN DECES D’UNE PERSONNE DE PLUS DE 2 ANS OUI NON

si non: continuer avec "AUTRES SYMPTOMES"

MAUX DE POITRINE, MAUX DE COTES OUI NON

Durée (H=heures; J=jours; S=semaines; M=mois; A=années): |__|__| |__|

Quand cela a-t-il commencé: |__|__| |__|__| |__|__|

Quand cela s’est-il terminé: |__|__| |__|__| |__|__|

Quelles parties du corps: ....

MAUX DE TETE OUI NON

Durée (H=heures; J=jours; S=semaines; M=mois; A=années): |__|__| |__|

Quand cela a-t-il commencé: |__|__| |__|__| |__|__|

Quand cela s’est-il terminé: |__|__| |__|__| |__|__|

Quelles parties de la tête: ....

Bourdonnements d’oreilles (Y=oui; N=non): |__|

Troubles visuels (Y=oui; N=non): |__|

MAUX DE VENTRE OUI NON

Durée (H=heures; J=jours; S=semaines; M=mois; A=années): |__|__| |__|

Quand cela a-t-il commencé: |__|__| |__|__| |__|__|

Quand cela s’est-il terminé: |__|__| |__|__| |__|__|

AUTRES SYMPTOMES OUI NON

Préciser lesquels: 1)..

2)..

<u>SYMPTOME 1:</u>

Durée (H=heures; J=jours; S=semaines; M=mois; A=années): |__|__| |__|

Quand cela a-t-il commencé: |__|__| |__|__| |__|__|

Quand cela s’est-il terminé: |__|__| |__|__| |__|__|

SYMPTOME 2:

Durée (H=heures; J=jours; S=semaines; M=mois; A=années): |__|__| |__|

Quand cela a-t-il commencé: |__|__| |__|__| |__|__|

Quand cela s'est-il terminé: |__|__| |__|__| |__|__|

SIGNES GENERAUX

Maigri au cours de la maladie (Y=oui; N=non): |__|

Déjà maigre au début de la maladie (Y=oui; N=non): |__|

Arrêté de manger au cours de la maladie (Y=oui; N=non): |__|

Etait très fatigué (Y=oui; N=non): |__|

Avait très soif durant la maladie (Y=oui; N=non): |__|

Paralysie du corps ou d'un membre (Y=oui; N=non): |__|

+ si oui: préciser quelles parties: ....

Perte de connaissance, coma (Y=oui; N=non): |__|

+ si oui quand au cours de la maladie (D=début; C=au cours; F=vers la fin; T=tout au long): |__|

Les mains ont-elles changé de couleur (Y=oui; N=non): |__|

Le corps a-t-il changé de couleur (Y=oui; N=non): |__|

Mangeait de la terre (Y=oui; N=non): |__|

Constipation (Y=oui; N=non): |__|

D'autres personnes ou d'autres enfants ont-ils eu les mêmes symptômes à la même période (Y=oui; N=non): |__|

+ si oui: dans quel village: ....

Goitre (Y=oui; N=non): |__|

+ si oui: depuis quand: |__|__| |__|__| |__|__|

Mauvaise vision dans la nuit (Y=oui; N=non): |__|

+ si oui: depuis quand: |__|__| |__|__| |__|__|

Emigré en Côte d'Ivoire (Y=oui; N=non): |__|

+ si oui: date de départ: |__|__| |__|__| |__|__|

date de retour: |__|__| |__|__| |__|__|

TRAITEMENTS RECUS POUR LA MALADIE ? **OUI** **NON**

Si oui: lesquels: ....

....

....

A PART DE SA MALADIE MORTELLE, EST-CE QUE LE DEFUNT A SOUFFERT D'UNE AUTRE MALADIE CHRONIQUE ? **OUI** **NON**

Si oui: quelle maladie: ....

....

....

SITUATION AU MOMENT DU DECES

∗ enfants décédés avant l'âge de 5 ans:

L'enfant a été sevré (Y=oui; N=non): |__|

∗ enfants décédés avant l'âge de 15 ans:

Marquer la date de vaccination:

BCG |__|__| |__|__| |__|__| Rougeole: |__|__| |__|__| |__|__|

DTCoq Polio (oral) ou DTCP (injectable) 2 |__|__| |__|__| |__|__|

∗ adultes décédé

état matrimoniale (C=célibataire; M=marié(e); D=divorcé(e); V=veuf/ve) |__|

∗ femmes décédées

nombre de grossesses: |__|__|

nombre d'accouchements: |__|__|

REMARQUES

....

....

....continuer au verso en cas de besoin.

FIN
