# Supplementary material for: An improved method for physician-certified verbal autopsy reduces the rate of discrepancy: experiences in the Nouna Health and Demographic Surveillance Site (NHDSS), Burkina Faso
Source: Popul Health Metr. 2011 Aug 4;9:34. doi: 10.1186/1478-7954-9-34 (PMC3160927; doi:10.1186/1478-7954-9-34)
Supplement: Additional file 2 — The restricted cause of death list based on ICD-10 used for the final physician coding. [file 1478-7954-9-34-S2.PDF]

Annex 2 :Restricted List of ICD 10 used for VA coding at Nouna HDSS

ICD10	full name	CodeCRSN
A00	Cholera	5
A01	Typhus fever	109
A05	Acute diarrhoea	3
A06	Chronic diarrhoea (ICD10: Amoebiasis unspecified)	251
A07	Dysentery	4
A09	Diarrhoeal disease	3
A15	Pulmonary Tuberculosis	105
A30	Leprosy	200
A33	Tetanus (neonatal)	106
A35	Tetanus (other)	107
A37	Whooping cough	102
A39	Meningitis	103
A71	Trachoma	208
A74	Conjunctivitis (Chlamydia)	12
A80	Poliomyelitis	110
A82	Rabies	11
A95	Yelow fever	104
B01	Chicken pox	14
B05	Measles	101
B16	Hepatitis	22
B20	AIDS	221
B26	Mumps	15
B50	Malaria (confirmed) / ICD10: Plasmodium falciparum malaria with cerebral complications	1
B54	Malaria (Unspecified / ICD10: Fever in endemic malaria areas)	1
B54	Fever (ICD10: Fever in endemic malaria areas)	2
B56	Trypanosomiasis	204
B65	Bilharziasis	220
B72	Guinea worm disease / Dracunculosis	239
B73	Onchocerciasis	209
B74	Filariasis	216
B35.0	Tinea barbae and Tinea capitis	240
C02	Malignant neoplasm:Tongue	278
C22	Malignant neoplasm: Liver unspecified	280
C26	Cancer digestive system	284
C39	Cancer airways	281
C50	Cancer of breast(unspecified)	282
C55	Malignant neoplasm genital tract	283
C71	Malignant neoplasm: Brain	279
D53	Anaemia	249
E07	Goitre	214
E14	Diabetes	203
E43	Malnutrition (Unspecified)	223
E50	Nocturnal blindness	211
F10	Mental disorder due to alcohool use	248
F99	Mental disorder (unspecified)	226

G40	Epilepsy	225
G83	Paralysis	217
H10	Conjunctivitis (unspecific)	12
H25	Cataract	212
H40	Glaucoma	213
H54	Blindness	210
H57	One-eyed (ICD10: other eye affections)	232
H91	Deafness	231
I10	Hypertensive heart and renal disease unspecified	245
I21	Myocardial infarction	18
I38	Heart disease	243
I**20**	Other forms of heart disease	205
I61	Cerebrovascular disease	19
I84	Haemorrhoid	207
J05	Laryngitis	25
J11	Flu (unspecific)	26
J18	Pneumonia	8
J20	Bronchitis	7
J45	Asthma	241
J81	Acute pulmonary oedema	27
K46	Hernia	219
K51	Bleeding of the digestive system	28
K56	Intestinal obstruction	21
K60	Anus cleft	229
K74	Cirrhosis of liver (Unspecified)	242
M25	Rheumatism	206
N00	Disease of the urinary system	246
N04	Nephrotic syndrome	247
N46	Male sterility / female sterility	238
N70	Inflammatory diseases of female pelvic organs	13
N71	Inflammatory diseases of the female pelvic organs	304
N73	Other inflammatory disease of the pelvic	310
N97	Female sterility	311
O06	Unspecified abortion	300
O15	Eclampsia (unspecified) (including pre existing hypertensive disorders)	307
O30	multiple pregnancy: Twin	308
O46	Antepartum haemorrhage	306
O49	Other antepartum causes	366
O79	Obstructed labour	302
O86	Puerperal infection	309
O99	Other postpartum causes	376
P00	Perinatal death affected by maternal disorders	405
P95	Stillbirth	400
P03	Asphyxia(fetus and newborn affected by complications of labour and delivery, unspecified)	406
P07	Premature, low birth weight	401
P39	Umbilicus infection	407
P51	Umbilicus haemorrhage	408
P59	Neonatal jaundice (unspecified)	403
P92	Malnutrition (neonatal)	404
P99	Other unspecified neonatal diseases	499
Q99	Congenital malformation (unspecified)	402
R02	Gangrene	250

R06	Hiccup	202
X00	Injury	10
X08	Poisoning (self harm)	20
X09	Food borne poisoning	20
X10	Snake bite	23
X11	insect bite	24
Y01	Homicide	900
Y02	Suicide	901
Z95	Maternal complications (unspecified)	389
Z98	Undetermined cause of death	999
Z99	No information available	998
_00	Convulsions	9
_00	Poisoning (unspecific)	20
_00	other acute disease	96
_00	acute disease (unspecific)	99
_00	Other preventable disease	196
_00	Preventable disease (unspecific)	199
_00	Jaundice (Unspecified)	222
_00	Wound	224
_00	Dizziness	228
_00	Tooth pain	230
_00	Ears infection	233
_00	Joints pain	235
_00	Haematuria	244
_00	Cancer skin and	285
_00	Other cancers	286
_00	Unspecified cancers	289
_00	Other chronic diseases	296
_00	Chronic disease unspecified	299
_00	Vaginal bleeding	301
_00	Vaginal discharges	303
_00	Other maternal complications	386
_00	Other maternal disease	396
_00	Maternal disease (unspecified)	399
_00	Other neonatal diseases	496
_00	Non applicable	997
